# A convolutional neural network for common coordinate registration of high-resolution histology images

**DOI:** 10.1093/bioinformatics/btab447

**Published:** 2021-06-15

**Authors:** Aidan C Daly, Krzysztof J Geras, Richard Bonneau

**Affiliations:** Center for Computational Biology, Flatiron Institute, New York, NY 10010, USA; Center for Data Science, New York University, New York, NY 10011, USA; Department of Radiology, Grossman School of Medicine, New York University, New York, NY 10016, USA; Center for Computational Biology, Flatiron Institute, New York, NY 10010, USA

## Abstract

**Motivation:**

Registration of histology images from multiple sources is a pressing problem in large-scale studies of spatial -omics data. Researchers often perform ‘common coordinate registration’, akin to segmentation, in which samples are partitioned based on tissue type to allow for quantitative comparison of similar regions across samples. Accuracy in such registration requires both high image resolution and global awareness, which mark a difficult balancing act for contemporary deep learning architectures.

**Results:**

We present a novel convolutional neural network (CNN) architecture that combines (i) a local classification CNN that extracts features from image patches sampled sparsely across the tissue surface and (ii) a global segmentation CNN that operates on these extracted features. This hybrid network can be trained in an end-to-end manner, and we demonstrate its relative merits over competing approaches on a reference histology dataset as well as two published spatial transcriptomics datasets. We believe that this paradigm will greatly enhance our ability to process spatial -omics data, and has general purpose applications for the processing of high-resolution histology images on commercially available GPUs.

**Availability and implementation:**

All code is publicly available at https://github.com/flatironinstitute/st_gridnet.

**Supplementary information:**

Supplementary data are available at *Bioinformatics* online.

## 1 Introduction

In the study of tissue pathology, spatial context matters. Tissues are composed of a variety of distinct cell types, often arranged in complex spatial patterns and interacting with neighbors via an intricate signaling network. As a result, measurements of cellular quantities of interest, such as mRNA or protein, may have very different interpretations depending on the precise location from which they were drawn. For this reason, bulk or single-cell methods for measuring the transcriptome or proteome, which can attain high yield of RNA/protein targets but require dissociation of the tissue, may not be ideal due to the substantial amount of contextual information they discard (Hwang *et al.*, 2018; Wang *et al.*, 2009). While computational approaches have been developed to map single-cell measurements back to coordinates in the original tissue (Edsgard *et al.*, 2018; Stuart *et al.*, 2019), recently developed high-throughput techniques for obtaining spatially resolved measurements of the transcriptome of *intact* tissue sections—either through highly multiplexed fluorescent *in situ* hybridization (FISH) (Sansone, 2019; Xia *et al.*, 2019) or solid-phase mRNA capture (Rodriques *et al.*, 2019; Ståhl *et al.*, 2016)—provide an attractive alternative due to their enhanced ability to observe pathological mechanisms at sub-cellular scales within their native environment. As technical advances continue to increase both their throughput and resolution (Asp *et al.*, 2020; Stickels *et al.*, 2020; Vickovic *et al.*, 2019), these so-called spatial ‘-omics’ methods promise an unprecedented view of complex tissue function and dysfunction.

While spatial -omics measurements of single tissue sections can be informative for applications such as patient-centered medicine, detecting trends at the level of organ, individual, or even population requires integrated analysis of data from multiple sources. Due to biological and technical variation, however, this cannot be accomplished by a simple overlay of results, and requires an initial *registration* step: a transformation that maps coordinates in one system to corresponding coordinates in another. When registering sequential images of a single tissue, one can rely on cellular landmarks, such as 4′,6-diamidino-2-phenylindole (DAPI)-stained nuclei, to define homography transforms that either minimize distance or maximize mutual information between images. When registering image data from multiple sources, we cannot expect to define a direct homography between samples, and instead define registration in terms of correspondence of higher-level features. One such approach involves segmenting tissue into distinct anatomical annotation regions (AARs) corresponding to conserved tissue types, effectively registering tissue to a common coordinate system and allowing for the comparison of like regions across samples (Rood *et al.*, 2019; Wang *et al.*, 2020).

This approach has inherent appeal in the field of spatial transcriptomics, in which image data are either explicitly segmented during downstream processing (FISH-based methods) or implicitly segmented by the experimental protocol (solid-phase capture methods). In FISH-based methods, channels reserved for cellular markers (such as DAPI) are used to segment cells, nuclei or other organelles so that mRNA/protein reads can be attributed to distinct entities, and thus contextualized by spatial co-occurrence. In this context, segmented entities can be assigned an AAR based on cell type, enabling comparison of similar entities across conditions. Solid-phase capture methods, such as spatial transcriptomics (ST) (Ståhl *et al.*, 2016) or 10× Genomics’ more recent Visium platform, load tissue onto slides specially printed with a regularly spaced array of discrete capture areas, or ‘spots’, each spanning a fixed area of tissue (100 µm diameter spots spaced 200 µm apart for ST; 55 µm diameter spots spaced 100 µm apart for Visium) and capable of capturing thousands (ST) to tens of thousands (Visium) of reads. The accompanying histology images are implicitly segmented by the locations of these spots, allowing the user to visualize the tissue microenvironment associated with each distinct measurement. Taking advantage of this inherent discretization, Maniatis *et al.* (2019)—in their study of amyotrophic lateral sclerosis in mouse and human spinal cords—and Maynard—in their morphological characterization of the human dorsolateral-prefrontal cortex—were able to assign AARs based on tissue type to each spot by visual inspection, allowing for the integration and quantitative analysis of gene expression data from multiple individuals.

As the volume of data generated by spatial -omics studies increases, reliance on manual annotation quickly becomes infeasible. Consequently, deep learning-based approaches to image registration become attractive alternatives for their potential to speed throughput and enable large-scale experimental designs. Histological image data, however, present several unique challenges that merit consideration and may hamper the application of existing deep learning approaches. Accurate registration often requires information at a sub-cellular scale in order to detect subtle textural differences indicating changes in cellular composition, and additionally at a global scale to detect large-scale patterns such as layering or symmetry. The size of the images being considered, however—potentially tens of millions of pixels—makes it difficult to maintain both high resolution and global awareness when training neural networks on commercially available hardware (Hekster *et al.*, 2020). On a fixed memory budget, this results in a tradeoff between global and local information for deep learning approaches. On the global end of this spectrum lie region segmentation approaches, which successively abstract images (through operations such as strided convolution or pooling) in order to partition them into distinct entities based on high-level characteristics. Biologically motivated architectures such as U-Net (Ronneberger *et al.*, 2015) additionally employ lower-level information into their segmentation output through the use of feed-forward connections, though this increase in network complexity commensurately limits the size of images that can be processed. One may address these limitations by reducing the complexity of the network (reducing the number of feature maps, increasing convolution stride, employing dilated convolutions) or downsampling the input images, thereby sacrificing an ability to detect local (sub-cellular) features, or by dividing the image into tiles and processing each independently, thereby sacrificing an ability to detect features spanning multiple tiles. In order to avoid the loss of salient information during segmentation, one must incorporate expert knowledge of the system being studied into the design of the network, such as the length scale of the largest and smallest features employed by pathologists during manual annotation. When one is primarily interested in discrete regions of interest (ROIs), such as ST spot locations, one may instead employ a more local approach by extracting image patches and independently applying an image classification convolutional neural network (CNN) to each. This ‘patch classification’ approach is popular in histopathology due to its ability to achieve high resolution with low overhead and was employed by Maniatis *et al.* (2019) in their development of the annotation tool Span (https://github.com/tare/span). The lack of global information, however, makes patch classification susceptible to local errors, particularly in regions of damaged tissue. This can be observed in the results of Tan *et al.* (2020), who apply a CNN architecture to identify cancer state in dissociated image patches from an ST study of prostate cancer biopsies. While some of these errors could be corrected by the application of *post hoc* denoising strategies for image segmentation such as conditional random fields (Lafferty *et al.*, 2001), a more powerful approach would be able to learn characteristics of tissue organization in an end-to-end manner, allowing the network to correct local predictions from a birds-eye view.

**Fig. 1.**
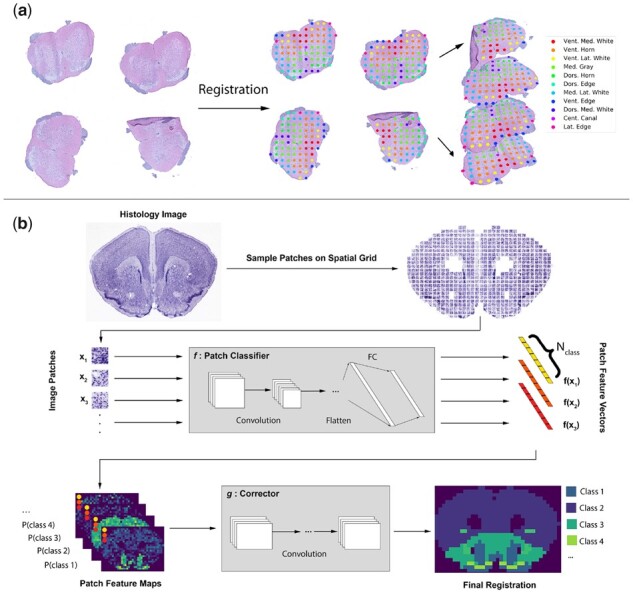
Common coordinate registration and the automation thereof by GridNet. (**a**) An example of common coordinate registration using mouse spinal cord tissue and 11 tissue classes. (**b**) Schematic of the proposed CNN architecture operating on a cross-section of mouse brain tissue. An image classification CNN *f* is employed to extract feature vectors from patches sampled from high-resolution histology images. These feature vectors serve as input to a second CNN *g* which is trained to perform semantic segmentation

Here, we present ‘GridNet’, a novel deep learning architecture for the registration of histology images to a common coordinate system. This network achieves an ability to model complex data by combining two CNNs: a classifier that is applied to full-resolution image patches extracted at sparsely sampled locations in the original histology image, and a segmentation network that operates on feature vectors extracted from each patch (Fig. 1b). The sparse feature extraction in the first stage of our network is similar to the approach of Tellez *et al.* (2021), though our network can be trained in an end-to-end manner, with the classification CNN learning cellular and sub-cellular features key to identifying tissue region and state, and the segmentation CNN learning global tissue patterns to correct for local errors. We explore several strategies for training GridNet, demonstrating approaches that address the differing demands of the component networks and are capable of being executed on a single GPU. We apply GridNet, as well as competing architectures for common coordinate registration, to two datasets with available gold-standard manual labels: a mouse brain section reference histology dataset obtained from the Allen Brain Atlas (ABA) (Wang *et al.*, 2020), and the mouse spinal cord ST dataset detailed in Maniatis *et al.* (2019). Using these data, we show that pure segmentation and pure classification approaches suffer, respectively, from limitations on model complexity and persistent local errors, while GridNet is able to leverage elements of both paradigms to attain consistently high-registration accuracy. Finally, we apply GridNet to a dataset of human dorsolateral prefrontal cortex (DLPFC) tissue processed with 10× Genomics’ recently released Visium spatial transcriptomics platform (Maynard *et al.*, 2021), which achieves high resolution by hexagonal packing of ST spots, in order to demonstrate the generality of our approach to new spatial systems. We believe our hybrid approach, for which we provide a publicly available Python implementation, presents a useful paradigm for general tasks in large-scale histology image processing, due to its demonstrated ability to blend information from both sub-cellular and global scales.

## 2 Materials and methods

### 2.1 Data specification

In this section, we discuss the manner by which image data were collected for this study, and how the points on the ‘registration grid’ from which we extract full-resolution image patches were chosen. For each tissue, image patches are sampled at the points in this grid and arranged into a five-dimensional array—height of registration grid, width of registration grid, height of patch, width of patch, number of image channels—that serves as an input to our tissue registration neural networks.

#### 2.1.1 ABA reference histology

**Fig. 2.**
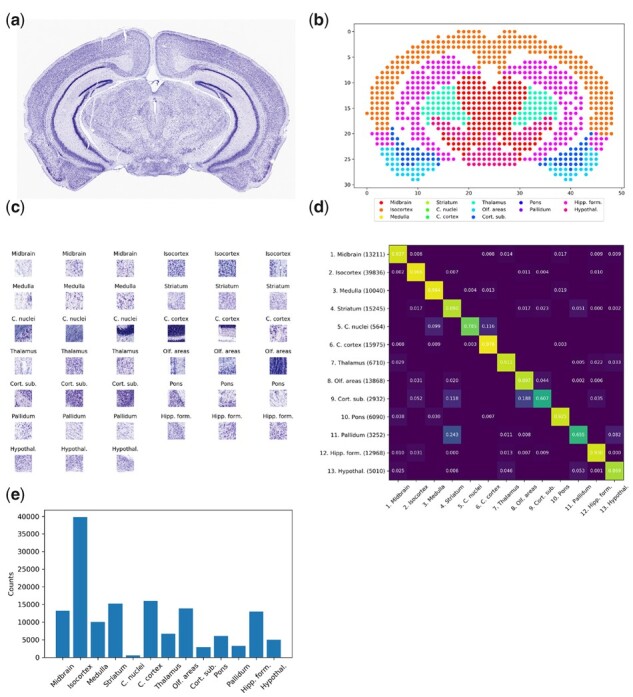
ABA reference histology dataset. (**a**) Representative histology image depicting a Nissl staining of a mouse brain coronal section. (**b**) Scatterplot displaying locations of corresponding ST spot centroids colored according to AAR annotation. (**c**) Three representative patches from each ontology region. (**d**) Adjacency matrix displaying the frequency with which patches of class [row] neighbor patches of class [column]. The number of instances of each class in the dataset is indicated in parentheses next to each row label in (**d**), as well as being displayed graphically in (**e**)

The ABA dataset, summarized in Figure 2, was constructed from 235 Nissl-stained sequential coronal sections of the mouse brain, which are publicly available at https://connectivity.brain-map.org/static/referencedata. For each image, we sampled patches from a two-dimensional (2D) Cartesian grid across the image, with a center-to-center distance of 192 pixels. This corresponds to ∼200 µm, which simulates the center–center distance in the 10× genomics standard ST array used by Maniatis *et al.* (2019). This sampling strategy required a 49 × 33-sized grid to span the tissue area of the largest section, and as such these dimensions were chosen as the fixed size of our simulated ST array. Using each position in the array as a centroid, we sampled patches of either 128 × 128 pixels or 256 × 256 pixels, depending on the input size of the architecture. For all patches, each image channel was separately normalized according to $x_{c}=(x_{c}-\mu_{c})/\sigma_{c}$, where $\mu_{\text{RGB}}=(0.485,0.456,0.406),\;\sigma_{\text{RGB}}=(0.229,0.224,0.225)$.

For each patch, an annotation was obtained by using the ABA’ Image Synchronization API (http://help.brain-map.org/display/api/Image-to-Image+Synchronization) to map the pixel coordinates of the patch centroid either to coordinates within a structural atlas of the mouse brain (http://atlas.brain-map.org/atlas?atlas=2) or to the slide background. Each position within the structural atlas is associated with a hierarchy of ontology terms, describing the location with increasing degrees of specificity. We chose the level-5 ontology term as label for each foreground patch, yielding 13 unique classes across the dataset. The complete dataset consisted of 149 943 foreground patches with 13 unique annotations across the 235 arrays. The number of patches belonging to each class is detailed in Figure 2(d). For experiments exploring optimal training strategies for GridNet, the patch size was set to 128 pixels, with 80% of the arrays used for training and 20% used for validation. For experiments comparing GridNet against competing methodologies for registration, the patch size was set to 256 pixels, with 70% of arrays were used for training, 10% for validation and 20% for testing.

#### 2.1.2 Maniatis mouse spinal cord ST

**Fig. 3.**
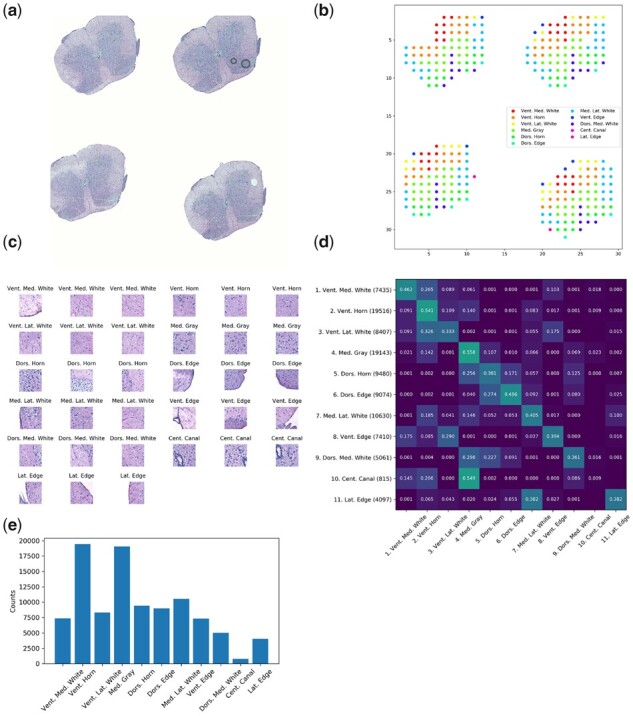
Maniatis mouse spinal cord ST dataset. (**a**) Representative histology image depicting HE staining of mouse spinal cord cross-section. (**b**) Scatterplot displaying locations of corresponding ST spot centroids colored according to AAR annotation. (**c**) Three representative image patches from each AAR, each displaying a (150 µm)^2^ region. (**d**) Adjacency matrix displaying the frequency with which patches of class [row] neighbor patches of class [column]. The number of instances of each class in the dataset is indicated in parentheses next to each row label in (**d**), as well as being displayed graphically in (**e**)

The Maniatis dataset, summarized in Figure 3, was constructed from 416 whole-slide images of hematoxylin and eosin (HE)-stained cross-sections of mouse spinal cord. Each of these imaged tissues had been processed using the Spatial Transcriptomics workflow described in Salmen *et al.* (2018), which employs 100 µm diameter mRNA capture probes arranged in a Cartesian grid at 200 μm center-to-center distance. The dimensions of all ST arrays are 35 rows by 33 columns.

Annotation files for each tissue section specifying the spatial location of each ‘foreground’ ST spot—spots which overlapped tissue area and attained a minimum number of mRNA reads—along with the corresponding manually assigned tissue labels (AARs) were obtained from Maniatis *et al.* (2019). Using these foreground ST spot locations as centroids, patches were sampled to cover a 256 × 256 pixel region, which corresponded to a physical area of ∼184 µm × 184 μm in each tissue. Due to variation in image resolution as a result of the data being collected from several distinct workflows, the width of the physical window size varied with a standard deviation of 34.5 μm across the dataset. For all patches, each image channel was separately normalized according to $x_{c}=(x_{c}-\mu_{c})/\sigma_{c}$, where $\mu_{\text{RGB}}=(0.485,0.456,0.406),\;\sigma_{\text{RGB}}=(0.229,0.224,0.225)$. The complete dataset consisted of 101 068 foreground patches with 11 unique classes across the 416 arrays. The number of patches belonging to each class is detailed in Figure 3(d). For all experiments, 70% of the arrays were used for training, 10% for validation and 20% for testing.

#### 2.1.3 Maynard human DLPFC Visium

**Fig. 4.**
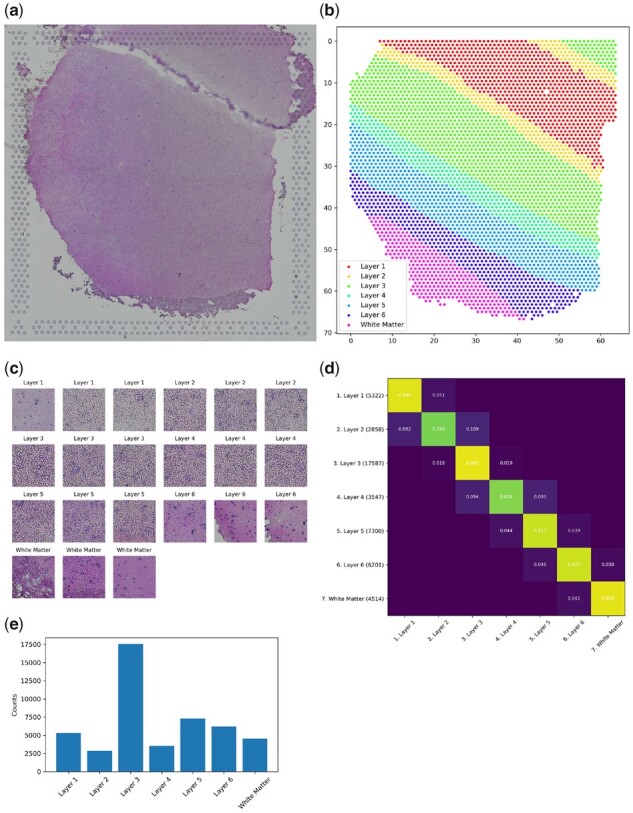
Maynard human DLPFC Visium ST dataset. (**a**) Representative histology image depicting HE staining of human DLPFC cross-section. (**b**) Scatterplot displaying hexagonally packed locations of corresponding Visium spot centroids colored according to AAR annotation. (**c**) Three representative image patches from each AAR, each displaying a (185 µm)^2^ region. (**d**) Adjacency matrix displaying the frequency with which patches of class [row] neighbor patches of class [column]. The number of instances of each class in the dataset is indicated in parentheses next to each row label in (**d**), as well as being displayed graphically in (**e**)

The Maynard dataset, summarized in Figure 4, was constructed from of 12 whole-slide images of HE-stained cross-sections of human DLPFC collected across three neurotypical patients. Each of these imaged tissues had been processed using 10× Genomics’ Visium Spatial Transcriptomics workflow, which employs 50 µm diameter mRNA capture probes arranged in a hexagonal grid at 100 µm center-to-center distance. The dimensions of all Visium array are 78 rows by 64 columns.

Annotation files for each tissue section specifying the spatial location of each foreground spot, along with manually assigned layer annotation, were obtained from Maynard. Using these foreground ST spot locations as centroids, patches were sampled to cover a 256 × 256 pixel region, which corresponded to a physical area 185 µm × 185 µm in each tissue. All three-channel image patches were normalized such that $\mu_{\text{RGB}}=(0.485,0.456,0.406),\;\sigma_{\text{RGB}}=(0.229,0.224,0.225)$. The complete dataset consisted of 47 329 foreground patches with 7 unique classes across the 12 arrays. The number of patches belonging to each class is detailed in Figure 4(d).

For experiments in this article, the data were divided into six equally sized folds for nested cross-validation. The following fold compositions were chosen, using sample numbers from the original publication: Fold 1 = (151 507, 151 508), Fold 2 = (151 509, 151 510), Fold 3 = (151 669, 151 670), Fold 4 = (151 671, 151 672), Fold 5 = (151 673, 151 674) and Fold 6 = (151 675, 151 676).

#### 2.1.4 Task specification

As the data being considered in this article are sampled according to regular grids—either Cartesian or hexagonal—we represent inputs to the registration model as a tensors of dimension $\left(H_{\text{ST}},W_{\text{ST}},H_{p},W_{p},C\right)$, where *H*_ST_ and *W*_ST_ represent the height and width of the ST grid, *H_p_* and *W_p_* represent the height and width of the patches and *C* represents the number of image channels. As all data considered in this article are square patches sampled from RGB images, we hold *H_p_* = *W_p_* and *C *=* *3.

Labels are represented as 2D tensors of dimension $\left(H_{\text{ST}},W_{\text{ST}}\right)$. Thus, for input *X* and label *Y*, $X_{i,j}$ yields the image patch (of dimension $H_{p}\times H_{p}\times 3$) corresponding to row *i*, column *j* in the ST array, while $Y_{i,j}$ yields the class label for that patch. All foreground patches are assigned a label between 1 and $N_{\text{class}}$—where $N_{\text{class}}$ represents the number of distinct foreground classes—while background patches are represented as zero-valued arrays and assigned a label of 0.

### 2.2 Model specification

In this section, we discuss several variants of the proposed ‘GridNet’ architecture for tissue registration, which is comprised of two component networks—a patch classification CNN and a segmentation CNN—that are connected and trained in an end-to-end manner. We provide an outline of the operation of the network on inputs constructed as outlined in the previous section, and additionally introduce a purely segmentation-based approach to tissue registration that will serve as a baseline for comparison of GridNet’s performance.

#### 2.2.1 GridNet architectures

The GridNet model is comprised of two components CNNs: patch classifier *f* and global corrector *g*. *f* accepts inputs of dimension $\left(H_{p},H_{p},3\right)$ and outputs a vector of predictions of length $N_{\text{class}}$:

(1) $$f(X_{i,j})\to Y\prime_{i,j},\;X_{i,j}\in {\left(0,1\right)}^{H_{p}\times H_{p}\times 3},\;Y\prime_{i,j}\in {\left(0,1\right)}^{N_{\text{class}}},$$

and *g* accepts inputs of dimension $\left(H_{\text{ST}},W_{\text{ST}},N_{\text{class}}\right)$ and outputs a matrix of the same dimension:

(2) $$g(Y\prime )\to Y\prime \prime ,\;Y\prime ,Y\prime \prime \in {\left(0,1\right)}^{H_{\text{ST}}\times W_{\text{ST}}\times N_{\text{class}}},$$

where *H*_ST_ and *W*_ST_ are the height and width (in patches) of the ST array, respectively.

The forward pass of the full model operates as follows: an input batch of shape $\left(B,H_{\text{ST}},W_{\text{ST}},H_{p},H_{p},3\right)$ (where *B* is the batch size) is flattened to a list of patches of dimension $\left(B\cdot H_{\text{ST}}\cdot W_{\text{ST}},H_{p},H_{p},3\right)$. Each patch ${\hat{X}}_{k}$ in this list is then passed through the patch classifier *f*, yielding an initial annotation $f({\hat{X}}_{k})$. The resulting tensor of dimension $\left(B\cdot H_{\text{ST}}\cdot W_{\text{ST}},N_{\text{class}}\right)$ is then reshaped to a tensor of dimension $\left(B,H_{\text{ST}},W_{\text{ST}},N_{\text{class}}\right)$. This tensor is then passed through the *g* network to obtain the final, identically sized registration map. Prior to output and error calculation, all background patches $\{X_{i,j}$ s.t. $\max(X_{i,j})=0\}$ are assigned class 0, while foreground patches are given a classification between 1 and $N_{\text{class}}$. By design, GridNet cannot mistakenly classify background patches as foreground or vice versa.

If the size of the input array is prohibitive for standard training by back-propagation, in which all intermediate activation states are stored in memory, the following strategy for gradient checkpointing is adopted: after obtaining the flattened patch list ${\hat{X}}_{k},$  $0<k\leq (B\cdot H_{\text{ST}}\cdot W_{\text{ST}})$,

1. Forward computation of mini-batch $f_{1}=f({\hat{X}}_{1}),\ldots ,f_{D}=f({\hat{X}}_{D})$. Save $f_{1},\ldots ,f_{D}$.
2. Repeat for all *D*-sized mini-batches ${\hat{X}}_{k},\ldots ,{\hat{X}}_{k+D}$ s.t. *k* > 1, $k+D\leq (B\cdot H_{\text{ST}}\cdot W_{\text{ST}})$.
3. Forward computation of $Y=g(f_{1},\ldots ,f_{B\cdot H_{\text{ST}}\cdot W_{\text{ST}}})$.
4. Backward computation of the gradient to update parameters of *g*.
5. Forward computation of mini-batch $f_{1}=f({\hat{X}}_{1}),\ldots ,f_{D}=f({\hat{X}}_{D})$.
6. Backward computation of the gradient to update parameters in *f* using only mini-batch ${\hat{X}}_{1},\ldots ,{\hat{X}}_{D}$. Save the gradients.
7. Repeat previous two steps for all mini-batches ${\hat{X}}_{k},\ldots ,{\hat{X}}_{k+D}$ s.t. *k* > 1, $k+D\leq (B\cdot H_{\text{ST}}\cdot W_{\text{ST}})$.
8. Average gradients for all $f_{1},\ldots ,f_{B\cdot H_{\text{ST}}\cdot W_{\text{ST}}}$ and apply parameter update.

This methodology effectively partitions the input array into mini-batches of size *D*, where *D* can be chosen based on the size of the input arrays and available GPU memory. For all experiments in this article using sufficiently large inputs (*H_p_* = 256), *D *=* *32 was chosen as the mini-batch size after experimentation on NVidia V100 32GB GPUs.

**Fig. 5.**
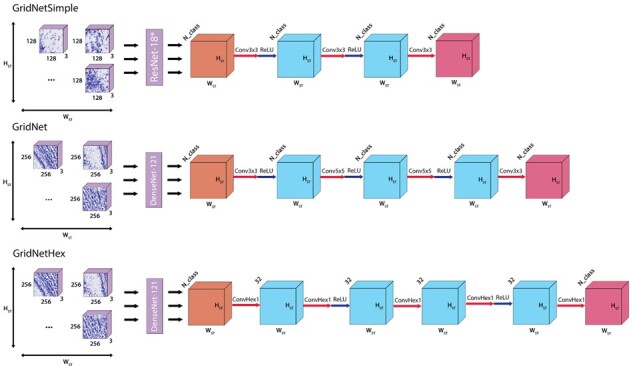
Three variant convolutional model architectures employed in image registration. ResNet18* indicates the modified ResNet architecture described in Section 2.2. Conv k×k layers indicate 2D standard convolutional layers with kernel size *k*, stride 1 and same padding. ConvHex*k* layers indicate 2D hexagonal convolutional layers with kernel size 1, stride 1 and same padding. ReLU arrows indicate a rectified linear unit activation function

Figure 5 details several variants of the GridNet architecture, defined by their choices for *f* and *g*. In GridNetSimple, a modified version of ResNet18 (He *et al.*, 2016) is employed for *f*, in which batch normalization layers are removed and the number of filters in each convolutional layer is reduced by a factor of four. In the more sophisticated GridNet and GridNetHex models, DenseNet-121 (Huang *et al.*, 2016) is employed for *f* instead. The choice of architecture for *g* is dependent upon the data being considered. When performing registration on either the ABA or Maniatis datasets, in which image patches are sampled according to a Cartesian grid, standard 2D convolutional kernels are employed to update the classification of a patch based on the classification of its neighbors. When considering the hexagonally sampled Maynard dataset, specially formulated hexagonal kernels are employed instead to accurately capture the six-neighborhood around each spot. In this study, this was accomplished through the use of hexagonal convolution operations as implemented in the HexagDLy (Steppa and Holch, 2019) extension to PyTorch.

#### 2.2.2 Segmentation architectures

As a baseline for comparison of the registration accuracy of GridNet, we formulated a modified version of ResNet-34 adapted to the registration task presented in this article (Supplementary Fig. S1). Inputs to this network were transformed from the inputs to GridNet, flattened along each ST dimension to yield a single ‘stitched’ image of dimension $\left(H_{\text{ST}}\cdot H_{p},W_{\text{ST}}\cdot H_{p},3\right)$. Though a choice of *H_p_* not equal to the center–center distance between spots will result in discontinuities in the input image, we have found that this does not affect network performance in any meaningful manner (Supplementary Fig. S7).

The ResNet-34 architecture was modified in two significant ways in order to process these data. First, the fully connected layers were discarded, and the final 2D adaptive average pooling layer was modified to output tensors of dimension $\left(H_{\text{ST}},W_{\text{ST}},N_{f}\right)$, where *N_f_* is the number of filters at this layer in the network. A final 1 × 1 2D convolution with stride 1, same padding and $N_{\text{class}}$ output filters was applied to this intermediate, yielding an output of dimension $\left(H_{\text{ST}},W_{\text{ST}},N_{\text{class}}\right)$. This enabled us to train the model on the same inputs and outputs as GridNet. The second modification was the reduction of the number of filters in each layer by a factor of four, which was required to fit even a single array from the ABA dataset into memory during training on a 32GB NVIDIA V100 GPU. As we had difficulty fitting even such a simple network in the memory of the GPU, we did not use U-Net style networks Ronneberger *et al.* (2015), which would require more memory.

### 2.3 Training regimens

For all models discussed in this study, the optimization criterion is the cross-entropy loss between the predicted class probabilities and their true labels.

When training the *f* network alone, the problem reduces to simple image classification. For an input batch of image patches $\hat{X}=\{{\hat{X}}_{0},\ldots ,{\hat{X}}_{B}\}$ (where all ${\hat{X}}_{b}\in {\left(0,1\right)}^{H_{p}\times H_{p}\times 3}$) and associated labels $\hat{Y}=\{{\hat{Y}}_{0},\ldots ,{\hat{Y}}_{B}\}$ (where all ${\hat{Y}}_{b}\in [1,\ldots ,N_{\text{class}}]$), the objective function is given as:

(3) $$\ell (\hat{X},\hat{Y})=\frac{1}{B}\sum_{b=0}^{B}-\text{log}\;\left(f({\hat{X}}_{b})[{\hat{Y}}_{b}]\right),$$

where $f({\hat{X}}_{b})[k]$ indicates the predicted probability of patch *b* belonging to class *k*, and ${\hat{Y}}_{b}$ indicates the index of the true class of patch *b*.

When training the *g* network alone, or both networks together, image patches and their tensors are arranged into 2D tensors as described in the previous section. For an input batch of image patch arrays $X=\{X_{0},\ldots ,X_{B}\}$ (where all $X_{b}\in {\left(0,1\right)}^{H_{\text{ST}}\times W_{\text{ST}}\times H_{p}\times H_{p}\times 3}$) and their associated labels $Y=\{Y_{0},\ldots ,Y_{B}\}$ (where all $Y_{b}\in {\left[1,\ldots ,N_{\text{class}}\right]}^{H_{\text{ST}}\times W_{\text{ST}}}$), the objective function is given as:

(4) $$\ell (X,Y)=\frac{1}{B}\sum_{b=0}^{B}\left[\sum_{\left(i,j)\in \text{FG}(Y_{b}\right)}-\text{log}\;\left(g{\left(X_{b}\right)}_{i,j}[Y_{b,i,j}]\right)\right],$$

where $\text{FG}(Y_{b})$ indicates the set of foreground patch coordinates in array *b*, $g{\left(X_{b}\right)}_{i,j}[k]$ indicates the predicted probability of the patch at location (*i*, *j*) in array *b* belonging to class *k* and $Y_{b,i,j}$ indicates the index of the true class of the patch at location (*i*, *j*) in array *b*.

Three competing training strategies were investigated for experiments on GridNet, all employing the Adam optimizer:

#### 2.3.1 Two-stage training

1. Train *f* on the set of all foreground patches in the training set using batch size $B\cdot H_{\text{ST}}\cdot W_{\text{ST}}$ patches for *E*_1_ epochs.
2. Fix parameters of *f*.
3. Train *g* using a batch size of *B* image arrays for *E*_2_ epochs.
4. Return the best-performing model on the validation set.

#### 2.3.2 At-once training

1. Train *f* and *g* simultaneously, using learning rate of *lr* for *g* and α·lr for *f*, using a batch size of *B* image arrays for *E* epochs.
2. Return the best-performing model on the validation set.

#### 2.3.3 Fine-tuning training

1. Initialize *f* and *g* with parameter values obtained from two-stage training approach. Save the state of the optimizer for the parameters in *g*.
2. Load the state of the optimizer for the parameters in *g*, and re-initialize the optimizer for *f*. Train *f* and *g* simultaneously, using learning rate of *lr* for *g* and α·lr for *f*, using a batch size of *B* image arrays for *E*_3_ epochs.
3. Return the best-performing model on the validation set.

### 2.4 Implementation details

All code is written in PyTorch (Paszke *et al.*, 2019) and is publicly available at https://github.com/flatironinstitute/st_gridnet/.

## 3 Results

In this section, we investigate several regimens for training the proposed GridNet model, then compare the registration performance attained by our model against competing approaches. We provide a schematic illustration of GridNet in Figure 1b, as well as summary figures of the three datasets considered in Figures 2–4.

### 3.1 Determining optimal training strategy

Due to the complexity of the GridNet architecture, we began by exploring training procedures for the network. For this analysis, we considered a simplified form of the architecture, GridNetSimple (Section 2.2, Fig. 5), which removes batch normalization layers from the patch classifier *f* [see Ioffe and Szegedy (2015) for a discussion of batch normalization in neural network training] in order to account for the different effective batch sizes of *f* and *g*. We trained the GridNetSimple architecture using the ABA reference histology dataset (Section 2.1.1, Fig. 2) with a patch size of 128 pixels. This smaller patch size was chosen in order to limit the size of input images and fit a batch size of *B *=* *2 arrays into GPU memory during training.

#### 3.1.1 Two-stage training

**Fig. 6.**
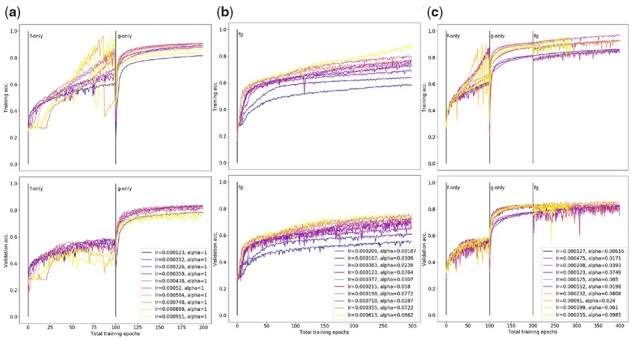
Registration accuracy attained by GridNetSimple architecture on ABA data under (**a**) two-stage (**b**) at-once and (**c**) fine-tuning training regimens (see Section 2.3). Each sub-plot shows training (top) and validation (bottom) accuracy as a function of training epoch, with a separate trace for each of 10 randomly sampled learning rates. Across all plots, ‘lr’ denotes learning rate for parameters of *g*, and ‘alpha’ denotes fraction of lr used as learning rate for parameters of *g*. Individual traces are color-coded from cool to warm according to the value of log(lr·α)

We first considered a two-stage training approach, in which we first train *f* alone on the set of all image patches in the training set for $E_{1}=50$ epochs, then fix the parameters of *f* and train *g* for $E_{2}=50$ epochs. Initially, we employed the same learning rate for both training phases, and repeated the fitting for 10 random samples from the interval log(lr)∼Uniform(−4,−3) (Fig. 6a). During these experiments, we ensured that training batches for *f* and *g* contained the same number of total image patches in order to remove batch information content as a confounding factor. In Figure 6a, we see that the best model obtained after training *f* alone attains 59% registration accuracy on the validation set, while the best model obtained after training *g* using fixed *f* attains 84% registration accuracy on the same validation set. We note that this large increase in validation set performance is observed whether the weights of *f* are initialized randomly or by pre-training on a corpus such as ImageNet (Deng *et al.*, 2009). Such pre-training merely reduces the number of training epochs required to attain the same performance in *f* (Supplementary Fig. S2). This finding is consistent with those in Raghu *et al.* (2019), who determined that transfer learning from ImageNet is more important for the correct initialization of the *magnitudes* of medical image network parameters, rather than their specific values. As a result, and due to the relatively large scale of the ABA dataset, we relied on models trained from randomly initialized weights for the remainder of this study.

#### 3.1.2 End-to-end training

While the results from the two-stage approach are encouraging, demonstrating the power of this hybrid classification approach even when both component models are extremely simple, training both components simultaneously is conceptually more appealing. Such an ‘end-to-end’ training approach would allow local learning rules to be updated in response to observed global patterns.

With this in mind, we next attempted training all model parameters at once from randomly initialized weights for *E *=* *300 epochs. While the parameters from *f* and *g* would be updated simultaneously under this regimen, it merited further consideration as to whether their parameters should change at the same rate. In the two-stage regimen, we held the learning rate for *f* and *g* to be equal for simplicity, but observe that higher learning rates lead to strong fluctuations in both training and validation set performance during optimization *f* (Fig. 6a). We hypothesized that this was due to the fact that parameters of *f* are coupled across all input image patches, thus impacting model predictions disproportionately when stepped at the same rate as the parameters of *g*. To address this, we allowed for the learning rate of *f* to be set at some fraction *α* of the learning rate for *g* during training. We found that this strategy gave the best results when α<0.1 (Supplementary Fig. S3), and as such sampled α∼Uniform(0,0.1) in subsequent analyses.

Accounting for this difference in learning rate, we see in Fig. 6(b) that the best-performing model trained in the at-once regimen (76% validation accuracy) still fails to match the validation set accuracy attained by the two-stage model, despite surpassing the accuracy attained by training *f* alone. In order to better guide the joint optimization in this large parameter space, we considered a new ‘fine-tuning’ approach to training, in which *f* and *g* are pre-trained following the two-stage regimen ($E_{1}=E_{2}=50$ epochs), then jointly trained as in the at-once approach for an additional $E_{3}=100$ epochs. In Fig. 6(c), we see that the best-performing model under this training regimen (86% validation accuracy) slightly outperforms the top models obtained by the two-stage approach, indicating the model may correct some errors arising from performing classification and segmentation in disjoint steps. While the at-once approach is appealing due to its simplicity, the relative success of the fine-tuning approach suggests that such an optimization is difficult even for simple models, and proper initialization of the parameters of *f* and *g* will be vital for success.

### 3.2 Registration of Cartesian ST data

Encouraged by our findings in the previous section, we increased the complexity of both *f* and *g* and applied the GridNet architecture (Section 2.2, Fig. 5) to both the ABA and the Maniatis datasets. For these analyses, we consider wider image patches (256 pixels) so that we may provide more information to our patch classification network and investigate the added benefit of the global segmentation layer under these conditions.

This increase in input size (≈1GB per tissue in each dataset) quickly exhausted the limits of memory on an NVidia V100 32GB RAM GPU during training with back-propagation, even when employing a batch size of just one array. In order to accommodate these large data, we implemented *gradient checkpointing*, described detail in Section 2.2.2., which allows the model to process input arrays in GPU-friendly mini-batches, and enables end-to-end training in instances where input arrays prohibitively large for standard back-propagation. In order to simulate batch sizes *greater* than one, we additionally implemented a *gradient accumulation* step, in which back-propagated gradients are summed over a number of input batches (five, in our experiments) before updating model parameters. This allows us to reduce noise in the parameter update step and aids in convergence to a local optimum. While multiple GPUs could be employed to alleviate some memory burden, particularly that associated with increased batch size, we have chosen to limit our experimentation to single GPUs in the interest of hardware accessibility.

We then sought to compare the performance of the improved GridNet against approaches based purely on classification or segmentation. For a pure classification approach, we applied the *f* network from GridNet (DenseNet-121) to all foreground image patches in the dataset independently. For a pure segmentation approach, we employed ‘ResNet-34-Seg’, a ResNet-34 architecture modified to produce registration maps from whole-slide images (see Section 2.2.3). Due to the size of the images, the number of filters in each layer of ResNet-34-Seg had to be reduced by a factor of four in order to accommodate a single input during training on a 32GB RAM GPU. For each model and dataset combination, we performed hyperparameter optimization by sampling 10 learning rates (and *α*’s, if applicable) according to log(lr)∼Uniform(−4,−3),α∼Uniform(0,0.1). From the five best-performing models on the validation set (Supplementary Table S1), we constructed an ensemble classifier by unweighted voting to yield an estimate of best-case performance. We additionally calculated the mean and standard deviation of accuracy and area under both the receiver operator curve (AUROC) and the precision-recall curve (AUPRC) across the component models to yield an estimate average-case performance.

In Table 1 (ABA) and Table 2 (Maniatis), we see that across both datasets, GridNet greatly outperforms both DenseNet-121 and ResNet-34-Seg in average-case and best-case registration accuracy, as well as per-class and macro average AUPRC and AUROC. Between the competing approaches, we see that registration by ResNet-34-Seg performs worse than registration by DenseNet-121 along the same metrics for both datasets, despite the fact that DenseNet-121 only operates on dissociated patches. We attribute this to the reduction in complexity of the ResNet-34-Seg architecture necessitated by the size of input images, and highlights the difficulty of performing segmentation for images of this size. We additionally note that the patch classification accuracy attained by DenseNet-121 is much lower in the Maniatis dataset than the ABA dataset. This is likely due to properties of the data themselves: the ABA dataset is a well-curated reference histology dataset, with highly consistent staining and sample orientation. The Maniatis dataset, on the other hand, presents variability in both tissue orientation and stain intensity (Supplementary Fig. S4), both of which can be confounding to image classification architectures. The predictive power of DenseNet may be enhanced by preprocessing images with stain normalization techniques, and additionally by the application of data augmentation to introduce robustness to orientation. Despite this variability, we see that the segmentation layer of GridNet adds substantially to the generalized registration accuracy on the Maniatis dataset (almost 12%), indicating that this approach is beneficial even when built on top of an imperfect classifier. While providing additional spatial context to *f* by doubling the size of the receptive field (*H_p_* = 512) does increase the registration performance of the model during the *f*-phase of training, we observed that the final registration accuracy attained by such models after two-stage training was identical to the models presented in this section under all investigated hyperparameters (Supplementary Fig. S5). To alleviate the quadratic increase in computational cost associated with increasing patch size, one may sample wider window and then downsample the image, affording greater context at decreased resolution. We found that increased window sizes of 512 and 1024 px do lead to increased accuracy in the *f* network on the Maniatis dataset, but still fail to meet the performance attained by our two-stage approach while also risking the loss of sub-cellular information (Supplementary Fig. S6). As such, we believe that more complex, multi-resolution approaches to patch classification may not warrant the increased memory burden and training time, as the neighborhood information learned by the *g* network appears to be much more important for final registration performance.

**Table 1.** Accuracy, AUROC and AUPRC attained by registration models on ABA independent test set

		Densenet-121		GridNet		ResNet-34-Seg	
		Ensemble	Mean (SD)	Ensemble	Mean (SD)	Ensemble	Mean (SD)
Accuracy		0.861	0.826 (4.4e−3)	0.912	0.892 (3.7e−3)	0.731	0.678 (3.08e−2)
AUPRC	1. Midbrain	0.905	0.861 (1.07e−2)	0.963	0.944 (6.04e−3)	0.641	0.552 (6.7e−1)
	2. Isocortex	0.978	0.973 (8.57e−4)	0.993	0.990 (1.51e−3)	0.962	0.942 (1.08e−2)
	3. Medulla	0.947	0.920 (9.09e−3)	0.984	0.976 (2.27e−3)	0.659	0.575 (4.81e−2)
	4. Striatum	0.863	0.837 (7.94e−3)	0.931	0.913 (1.08e−2)	0.709	0.612 (5.71e−2)
	5. Cerebellar nuclei	0.841	0.789 (2.34e−2)	0.914	0.787 (8.84e−2)	0.513	0.364 (6.08e−2)
	6. Cerebellar cortex	0.986	0.980 (1.84e−3)	0.994	0.991 (1.32e−3)	0.947	0.931 (5.66e−3)
	7. Thalamus	0.942	0.914 (4.28e−3)	0.979	0.967 (4.23e−3)	0.778	0.664 (8.88e−2)
	8. Olfactory areas	0.898	0.866 (8.00e−3)	0.948	0.938 (6.82e−3)	0.893	0.783 (1.58e−2)
	9. Cortical subplate	0.647	0.573 (6.06e−3)	0.747	0.696 (1.51e−3)	0.364	0.254 (5.46e−2)
	10. Pons	0.836	0.762 (3.75e−2)	0.937	0.898 (2.32e−2)	0.398	0.307 (3.60e−2)
	11. Pallidum	0.540	0.447 (2.49e−2)	0.667	0.564 (7.14e−2)	0.253	0.164 (3.36e−2)
	12. Hippocampal formation	0.936	0.911 (3.67e−3)	0.980	0.966 (8.53e−3)	0.882	0.822 (3.31e−2)
	13. Hypothalamus	0.860	0.794 (1.92e−2)	0.945	0.915 (1.13e−2)	0.481	0.363 (5.96e−2)
	Macro average	0.860	0.818 (0.149)	0.922	0.888 (0.128)	0.648	0.564 (0.254)
AUROC	1. Midbrain	0.988	0.981 (2.30e−3)	0.996	0.994 (8.79e−4)	0.935	0.921 (1.91e−2)
	2. Isocortex	0.988	0.985 (7.34e−4)	0.997	0.995 (1.27e−3)	0.980	0.970 (5.79e−3)
	3. Medulla	0.994	0.992 (1.37e−3)	0.998	0.998 (1.10e−3)	0.959	0.942 (8.00e−3)
	4. Striatum	0.978	0.971 (1.40e−3)	0.991	0.989 (2.61e−3)	0.943	0.915 (2.04e−2)
	5. Cerebellar nuclei	0.998	0.996 (1.32e−3)	0.999	0.998 (1.25e−3)	0.980	0.959 (9.38e−3)
	6. Cerebellar cortex	0.998	0.997 (2.71e−4)	0.999	0.999 (2.08e−4)	0.985	0.978 (2.93e−3)
	7. Thalamus	0.994	0.990 (1.33e−3)	0.998	0.997 (7.22e−4)	0.966	0.948 (1.36e−2)
	8. Olfactory areas	0.986	0.980 (7.38e−4)	0.994	0.993 (1.20e−3)	0.971	0.953 (3.93e−3)
	9. Cortical subplate	0.971	0.962 (3.22e−3)	0.983	0.981 (1.27e−3)	0.935	0.894 (1.98e−2)
	10. Pons	0.989	0.982 (4.08e−3)	0.996	0.995 (1.10e−3)	0.930	0.908 (1.36e−2)
	11. Pallidum	0.973	0.964 (4.71e−3)	0.988	0.984 (2.62e−3)	0.931	0.897 (1.36e−2)
	12. Hippocampal formation	0.991	0.987 (6.55e−4)	0.997	0.995 (1.25e−3)	0.977	0.960 (8.81e−3)
	13. Hypothalamus	0.992	0.987 (1.47e−3)	0.997	0.995 (4.77e−4)	0.947	0.928 (1.56e−2)
	Macro average	0.988	0.983 (1.09e−2)	0.995	0.993 (5.4e−3)	0.957	0.937 (2.99e−2)

*Note*: Results shown were calculated using the five best-performing models obtained during hyperparameter optimization using the validation set. ‘Ensemble’ denotes results obtained by ensemble classifier built by unweighted voting of the five best models, while mean and standard deviation (SD) for performance metrics are calculated using the independent predictions from said models. AUROC and AUPRC are reported for each class (one-vs-rest) and for macro average across all 13 classes.

**Table 2.** Accuracy, AUROC and AUPRC attained by registration models on Maniatis independent test set

		Densenet-121		GridNet		ResNet-34-Seg	
		Ensemble	Mean (SD)	Ensemble	Mean (SD)	Ensemble	Mean (SD)
Accuracy		0.691	0.666 (3.6e−3)	0.806	0.782 (8e−4)	0.665	0.622 (1e−2)
AUPRC	1. Ventral medial white	0.610	0.567 (4.64e−3)	0.851	0.826 (3.54e−3)	0.635	0.549 (3.0e−2)
	2. Ventral horn	0.851	0.836 (2.96e−3)	0.917	0.904 (2.18e−3)	0.827	0.792 (1.18e−2)
	3. Ventral lateral white	0.554	0.516 (1.024e−2)	0.789	0.752 (3.77e−3)	0.536	0.484 (1.21e−2)
	4. Medial gray	0.807	0.791 (5.88e−3)	0.896	0.877 (3.94e−3)	0.774	0.731 (1.68e−2)
	5. Dorsal horn	0.869	0.855 (3.03e−3)	0.883	0.863 (2.01e−3)	0.853	0.816 (5.28e−3)
	6. Dorsal edge	0.820	0.801 (1.02e−2)	0.890	0.874 (4.88e−3)	0.771	0.719 (8.66e−3)
	7. Medial lateral white	0.630	0.585 (1.169e−2)	0.853	0.824 (2.13e−3)	0.565	0.507 (1.5e−2)
	8. Ventral edge	0.730	0.703 (1.02e−2)	0.872	0.849 (4.30e−3)	0.615	0.562 (4.46e−3)
	9. Dorsal medial white	0.740	0.706 (1.05e−2)	0.881	0.854 (4.54e−3)	0.779	0.712 (2.67e−2)
	10. Central canal	0.795	0.795 (8.04e−3)	0.790	0.739 (1.88e−2)	0.758	0.694 (5.34e−2)
	11. Lateral edge	0.393	0.368 (4.75e−3)	0.606	0.560 (9.82e−3)	0.324	0.289 (2.51e−2)
	Macro average	0.709	0.684 (0.149)	0.839	0.811 (9.31e−2)	0.676	0.623 (0.153)
AUROC	1. Ventral medial white	0.939	0.930 (1.17e−3)	0.980	0.976 (1.17e−3)	0.934	0.918 (5.77e−3)
	2. Ventral horn	0.958	0.953 (9.41e−4)	0.976	0.971 (8.60e−4)	0.949	0.937 (3.33e−3)
	3. Ventral lateral white	0.934	0.927 (1.49e−3)	0.975	0.970 (4.08e−4)	0.934	0.922 (3.43e−3)
	4. Medial gray	0.950	0.945 (1.60e−3)	0.972	0.967 (6.87e−4)	0.944	0.932 (4.01e−3)
	5. Dorsal horn	0.972	0.970 (1.45e−3)	0.980	0.976 (1.00e−3)	0.969	0.960 (1.45e−3)
	6. Dorsal edge	0.973	0.970 (1.25e−3)	0.985	0.983 (6.29e−4)	0.962	0.952 (2.49e−3)
	7. Medial lateral white	0.931	0.920 (2.56e−3)	0.974	0.968 (9.00e−4)	0.916	0.900 (4.10e−3)
	8. Ventral edge	0.973	0.969 (1.76e−3)	0.988	0.985 (7.72e−4)	0.963	0.957 (8.58e−4)
	9. Dorsal medial white	0.961	0.955 (3.27e−3)	0.986	0.982 (1.17e−3)	0.971	0.959 (3.13e−3)
	10. Central canal	0.978	0.976 (2.80e−3)	0.984	0.984 (7.47e−4)	0.977	0.973 (6.73e−3)
	11. Lateral edge	0.951	0.947 (1.01e−3)	0.974	0.969 (7.91e−4)	0.939	0.928 (4.75e−3)
	Macro average	0.956	0.951 (1.86e−2)	0.980	0.976 (6.7e−3)	0.951	0.940 (2.13e−2)

*Note*: Results shown were calculated using the five best-performing models obtained during hyperparameter optimization using the validation set. ‘Ensemble’ denotes results obtained by ensemble classifier built by unweighted voting of the five best models, while mean and standard deviation (SD) for performance metrics are calculated using the independent predictions from said models. AUROC and AUPRC are reported for each class (one-vs-rest) and for macro average across all 11 classes.

**Fig. 7.**
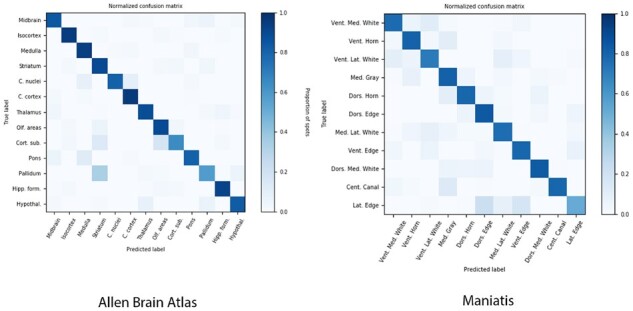
Confusion matrices for GridNet model on (left) ABA and (right) Maniatis datasets. Results shown were calculated on the held-out test set using the best-performing model on validation set. Rows are normalized such that each entry contains the proportion of patches with true label [row] that are classified as [column]

We note that in both datasets, GridNet displays uniformly high AUROC across all one-vs-rest class predictors and high AUPRC across most (Tables 1 and 2), though the macro average AUPRC is hampered by relatively poor performance on select classes (cortical subplate and pallidum in ABA; ventral lateral white, central canal and lateral edge in Maniatis). To understand this, we generated confusion matrices for each dataset to get a more granular view on the types of errors being made by GridNet (Fig. 7). In both ABA and Maniatis datasets, we notice that there is substantial overlap between the large off-diagonal elements of the confusion matrices, which indicate persistent errors mistaking one class for another, and the large off-diagonal elements in the class adjacency matrices (Figures 2d and 3d). This suggests that many of the errors made by GridNet involve mistakes between classes that are frequently neighbors, such as pallidum and striatum in ABA, and central canal and medial gray matter in Maniatis. Furthermore, we note that GridNet most frequently misclassifies patches belonging to rare classes, such as cerebellar nuclei, cortical subplate and pallidum in ABA, and central canal and lateral edge in Maniatis. This suggests that in such boundary cases, GridNet may default to making predictions close to the class prior (the frequency of each class in the training data) in order to minimize the chance of making costly mistakes.

**Fig. 8.**
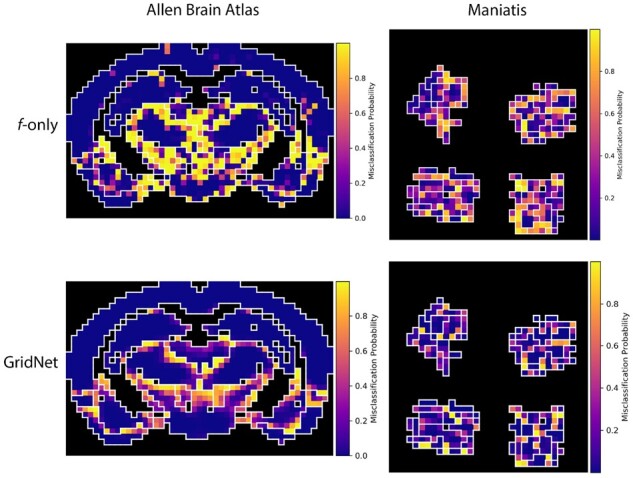
Misclassification probability density maps for representative images from (left) ABA and (right) Maniatis mouse spinal cord datasets. Each pixel is colored according to 1 − *P*(correct), as estimated by either the full GridNet model (top row) or the *f* component only (bottom row), for the corresponding image patch. Patches indicating slide background are rendered in black, and class boundaries are denoted by white outlines

These prevalent boundary errors may be influenced by the presence of noisy labels, resulting from the inherent difficulty of drawing hard boundaries on continuously varying tissue. In Figure 8, we visualized the probability of misclassifying each patch—1−P(correct), where P(correct) is calculated using the classification probabilities output by the best-performing model and the index of the true label—across representative tissue arrays from each dataset. For predictions made using the *f* network only, we see a rather uniform distribution of misclassification probability density, as well as a ‘spiking’ behavior where patches are far more likely to be misclassified in regions of mono-class tissue when compared to predictions made using the full network. In Supplementary Fig. S8, we demonstrate that the full network is best able to address such errors when a majority of the neighboring patches are correctly classified, confirming that the *g* network demonstrates the ‘corrector’ behavior that we have posited. Using the full network, we see that the misclassification density is greatly reduced overall, and the remaining uncertainty is highly concentrated near boundaries between tissue classes, or boundaries between foreground and background patches. This suggests that GridNet is able to learn from the context of surrounding tissue to correct errors, and when properly trained, persistent error may simply reflect inescapable error in tissue labeling.

### 3.3 Registration of non-Cartesian ST data

Finally, we sought to demonstrate the utility of GridNet for spatial data which are sampled in a non-Cartesian grid—namely, the recently released Visium ST platform from 10× Genomics. The hexagonal spot array used in this assay yields greater packing density (see Fig. 4b), increasing spatial resolution, but precludes the use of standard 2D-convolutional operations in *g*. Our GridNet approach can still be applied to these data through the modification of *g* to employ *hexagonal* convolution operations, which take into account the true six-neighborhood of each spot (see Section 2.2.1). Using this modified form of GridNet, GridNetHex (Section 2.1, Fig. 5), we assessed the registration performance on the Maynard human DLPFC Visium dataset (Fig. 4). While the increased resolution of the Visium arrays yields a corresponding increase in the number of patches (4992 spots per array in Visium compared to 1155 in standard ST), the small number of tissues processed by this study (12) limits the amount we can expect to learn from these data alone. As a result, we applied two-stage training with $E_{1}=E_{2}=100$ training epochs in each phase to minimize over-fitting, and initialized the parameters of *f* with the optimal values obtained from our ABA experiment (Section 3.2) in order to speed convergence. Furthermore, for this analysis we chose to assess the generalized performance of our model using 6-fold nested cross-validation, rather than employing a fixed train-validation test partition as we have for larger datasets. During nested cross-validation analysis, each fold was held out as a test set exactly once, while hyperparameters were separately optimized for each fold by performing cross-validation on the remaining five folds. For each fold of the cross-validation analysis, five parameters were drawn from the ranges log(lr)∼Uniform(−4,−3),α∼Uniform(0,0.1). The models with the best validation performance for each cross-validation fold were used to generate an ensemble predictor for the corresponding test fold.

In Table 3, we see that across the six folds, the ensemble network built from the full GridNetHex model yields small gains in registration accuracy over the ensemble network built from the *f* network alone. The negligible magnitude of improvement over patch classification alone suggests, unsurprisingly, that more samples will be needed in order for the *g* network to learn global patterns that can refine local predictions. This is supported by the largely identical AUROC and AUPRC performance, though these metrics do indicate that the model performs worst on Layers 2 and 4, which are the most infrequent and least contiguous tissues (Fig. 4d). The relatively low accuracy of the patch classifier can be further understood by examining the quality of the Maynard data (Fig. 4c), which were collected using a confocal microscope instead of the higher-resolution slide scanner employed in the other datasets. This resulted in lower-resolution patch images in which few sub-cellular features besides the size and placement of nuclei can be visibly distinguished. With higher-resolution imaging, as well as expansion of the number of tissue being trained upon, we will be able to increase the generalized accuracy of our DLPFC registration network. Despite this, our preliminary analysis demonstrates how GridNet may be applied to the registration of data collected from the Visium platform.

**Table 3.** Performance of ensemble GridNetHex models on held-out test folds during nested cross-validation analysis of the Maynard dataset

		*f*	*g*
Accuracy		0.544	0.564
AUPRC	1. Layer 1	0.652	0.661
	2. Layer 2	0.287	0.227
	3. Layer 3	0.665	0.655
	4. Layer 4	0.173	0.191
	5. Layer 5	0.370	0.386
	6. Layer 6	0.399	0.446
	7. White matter	0.826	0.843
	Macro average	0.482	0.487
AUROC	1. Layer 1	0.841	0.849
	2. Layer 2	0.746	0.711
	3. Layer 3	0.781	0.763
	4. Layer 4	0.704	0.704
	5. Layer 5	0.778	0.781
	6. Layer 6	0.794	0.804
	7. White matter	0.952	0.960
	Macro average	0.799	0.796

*Note*: Each column details the performance of an ensemble model created using the best-performing models found during cross-validation on the remaining five folds. Sub-columns distinguish between the performance of the ensemble created with the *f* network only or the full model (*g*). AUPRC and AUROC are reported for each class (one-vs-rest) and for macro average across all seven classes.

## 4 Discussion

We present GridNet, a novel deep learning network for common coordinate registration of high-resolution histology images. GridNet balances the need for sub-cellular resolution and awareness of spatial context by applying a hybrid classification-segmentation architecture to high-resolution image patches sampled sparsely across the tissue. Using this architecture, our models can predict tissue type for ROIs in large tissues while respecting the memory limits of commercially available GPUs.

Through experimentation on both simulated and experimental spatial datasets, we demonstrated that the hybrid classification-segmentation architecture consistently out-performed competing approaches in the registration of spatial transcriptomic data. Compared to a pure segmentation approach based on ResNet-34 (which required substantial reduction in complexity in order to process the histology images being considered) and a pure classification approach based on DenseNet-121, we saw significant reduction in error across the tissue, and notably a reduction in the chance of misclassifying tissue within a contiguous region. Most remaining error was concentrated on class boundaries, reflecting the difficulty of assigning discrete labels to a continuously varying input. Near such boundaries, predictions made by GridNet or competing registration architectures may skew toward the class prior in an attempt to minimize average-case misclassification error. While this behavior may be expected even in the presence of perfect training labels, and may be addressed in future iterations by weighting the loss function based on observed class frequency, the presence of noisy labels may greatly exacerbate it. The incorporation of replicate labeling data gathered from independent pathologists may help in the reduction of labeling noise, though a robust solution to this problem will require modification of the training objective function to explicitly model label noise, rather than assuming a gold standard. Methods for training neural networks in the presence of noisy labels is an active area of research (Song *et al.*, 2020; Vahdat, 2017; Zhang and Sabuncu, 2018), and may benefit future iterations of the GridNet model.

While the hybrid classification-segmentation approach is capable of attaining high-registration accuracy, the complex nature of the network architecture demands specific consideration during training. We found that the coupling of parameters in the *f* network across all input image patches yielded a different ideal learning rate from *g*, adding an additional hyperparameter during model training that must be tuned to the data at hand. Furthermore, we determined that pre-training of both component networks prior to end-to-end fine-tuning is instrumental in attaining high performance. Through experimentation on the ABA dataset, we developed a two-stage pre-training approach that yielded consistently strong results: parameters of local predictor *f* are initialized with values that yield the highest performance on the stand-alone task of foreground patch classification, then parameters of the global corrector *g* are optimized using *f* as a fixed feature extractor. While this method was sufficient to produce models exceeding the performance of competing approaches, we hope that further study of the network properties will reveal end-to-end training strategies that are more robust to initial parameter values.

In our brief analysis of ST data gathered with 10× Genomics’ Visium platform, we demonstrated that the GridNet paradigm could be applied when patches are sampled in non-Cartesian grids. The logical extension is the application of GridNet to data in which patches are sampled in an irregular manner. In FISH-based ST data, for example, the ROIs are not defined by the fixed grid capture probes, but instead by the locations of cell nuclei. One may imagine *f* being applied to classify extracted nuclei into discrete cell types, and *g* leveraging information from neighboring cells to correct said predictions. This could be accomplished by modeling nuclei as an undirected graph with edges weighted by Euclidean distance between them, and converting *g* to a graph CNN to aggregate information from neighbors. Such a formulation can be thought of as a super-set of GridNet, although standard or hexagonal convolutional operations should be used for *g* when possible due to their relative efficiency.

Finally, we are interested in developing future iterations of GridNet that leverage data from multiple modalities at once. As the ST experimental workflow produces paired measurements of both histology and gene expression, we may seek to combine information from these two modalities in our patch prediction network. A recent study by Tan *et al.* (2020) explored how integration of these two modalities within a single classifier may yield increased ability to predict tissue when considering ST spots independently, but did not consider how surrounding context could be used to refine these predictions. We believe that future iterations of GridNet could build off of such multi-modal feature extractors to build even stronger models for tissue registration. Furthermore, such multi-modal models could be adapted to the task of predicting one data modality from the other. Using a set of ST data, models could be trained to predict the expression of key genes from histology data alone, allowing researchers to predict expensive gene expression measurements from inexpensive histology images. Our architecture is well adapted for multi-modal contexts where orthogonal sensors, assays or tests are applied sparsely to an instance with a corresponding high-resolution image. Here we focus on spatial genomics, but applications for such a context and resolution hierarchy abound. Thus, we believe the GridNet paradigm will be a useful and generalizable approach to making predictions on a variety of spatial biological data, and building upon the implementation presented in this article, can scale with the increasing size and resolution of spatial data being acquired today.

*Financial Support*: none declared.

*Conflict of Interest*: none declared.

## Supplementary Material

btab447_Supplementary_Data
