# Supplementary material for: A convolutional neural network for common coordinate registration of high-resolution histology images
Source: Bioinformatics. 2021 Jun 15;37(22):4216–26. doi: 10.1093/bioinformatics/btab447 (PMC9502165; doi:10.1093/bioinformatics/btab447)
Supplement: btab447_Supplementary_Data [file btab447_supplementary_data.pdf]

Supplementary Material: A convolutional neural network for common coordinate registration of high-resolution histopathology images

Aidan C. Daly*[1], Krzysztof J. Geras†[3,2], and Richard A. Bonneau‡[1]

[1]Center for Computational Biology, Flatiron Institute, U.S.A.
[2]Center for Data Science, New York University, U.S.A.
[3]NYU Grossman School of Medicine, U.S.A.

March 15, 2021

*adaly@flatironinstitute.org
†k.j.geras@nyu.edu
‡rbonneau@flatironinstitute.org

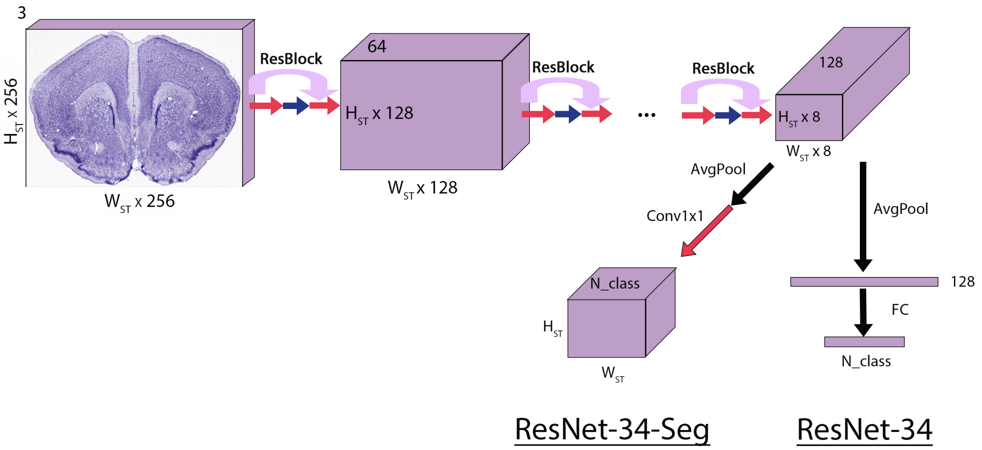

Figure 1: Modifications applied to ResNet-34 in order to create ResNet-34-Seg for common coordinate registration of high-resolution histopathology images. “ResBlock” connections indicate the residual block comprising ResNet, which contain skip connections in order to feed foward low-level image information. “AvgPool” arrows indicates average pooling layers, while “FC” arrows indicate fully-connected linear layers.

Model	ABA		Maniatis	
	lr	α	lr	α
GridNet	1.857e-4	5.618e-2	6.685e-4	3.527e-2
	8.106e-4	5.251e-2	8.052e-4	6.921e-2
	3.054e-4	8.632e-2	4.127e-4	1.254e-2
	2.379e-4	6.889e-2	6.77e-4	9.795e-2
	2.389e-4	2.835e-2	3.33e-4	1.657e-2
ResNet-34-Seg	1.029e-4		3.438e-4	
	2.559e-4		1.256e-4	
	1.664e-4		1.017e-4	
	2.579e-4		4.724e-4	
	4.199e-4		1.257e-4	

Table 1: Highest performing hyperparameter combinations from validation study of GridNet and ResNet-34-Seg on Allen Brain Atlas and Maniatis datasets.

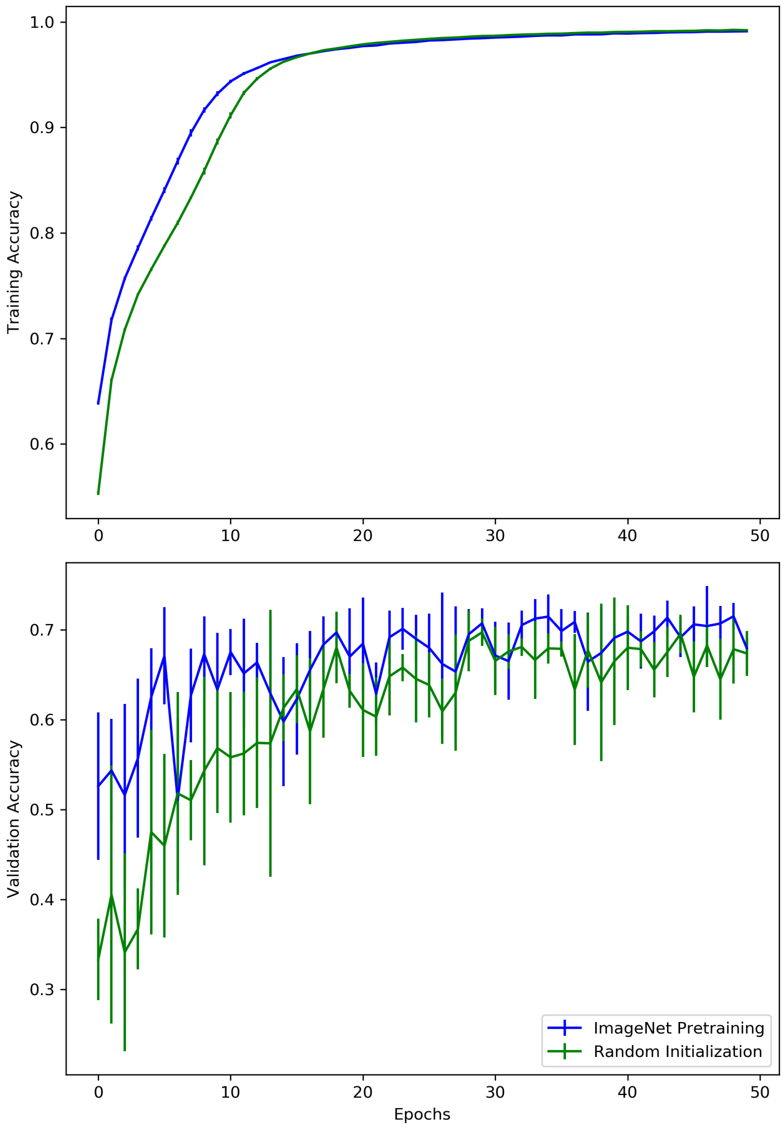

Figure 2: Registration accuracy attained by training ResNet-18 on image patches from the Allen Brain Atlas dataset under two training regimens: random weight initialization, and weight initialization by pre-training on ImageNet. Each training regimen was repeated five times using the Adam optimizer with a learning rate of 0.001. Mean training/validation accuracy is plotted for each regimen, with error bars denoting standard deviation.

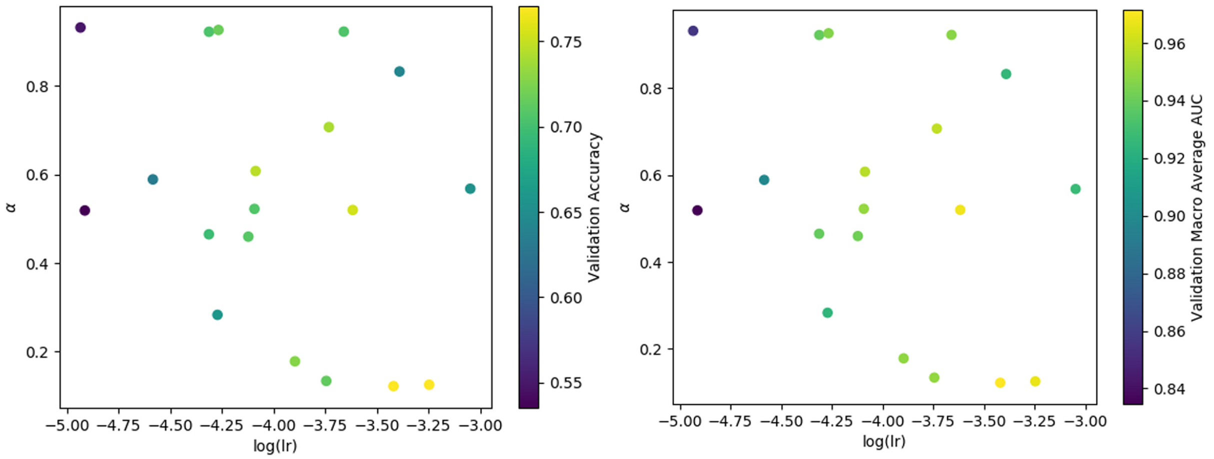

Figure 3: Effects of learning rate and alpha parameters on training of GridNetSimple registration model on Allen Brain Atlas dataset using the at-once training regimen. Each point represents a separate training from randomly initialized parameters, with points colored according to either the accuracy (left) or macro average ROC-AUC (right) attained on the validation set.

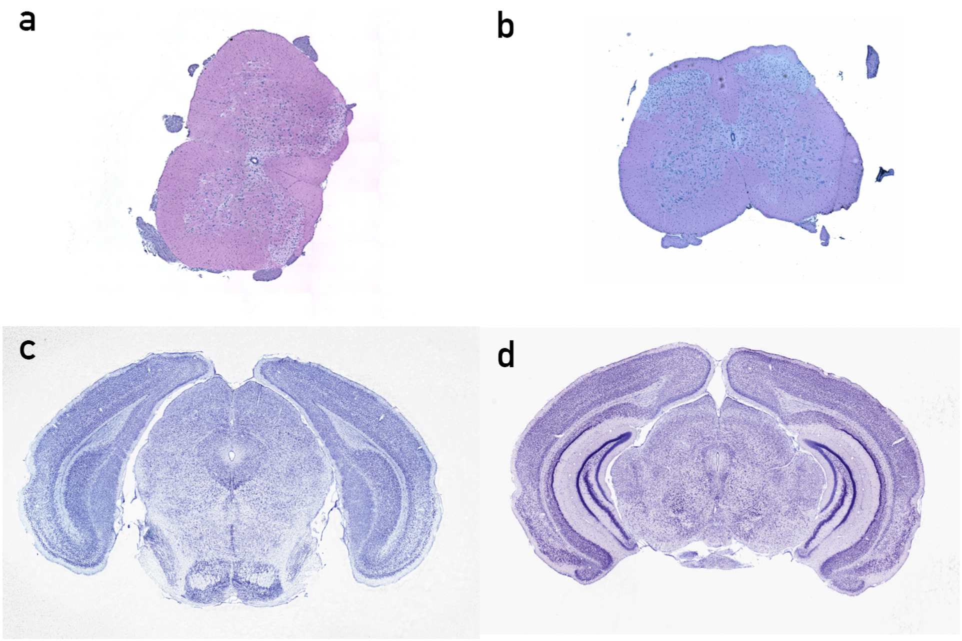

Figure 4: Selected images exemplifying variation in staining and orientation in the Maniatis (a,b) and Allen Brain Atlas (c,d) datasets.

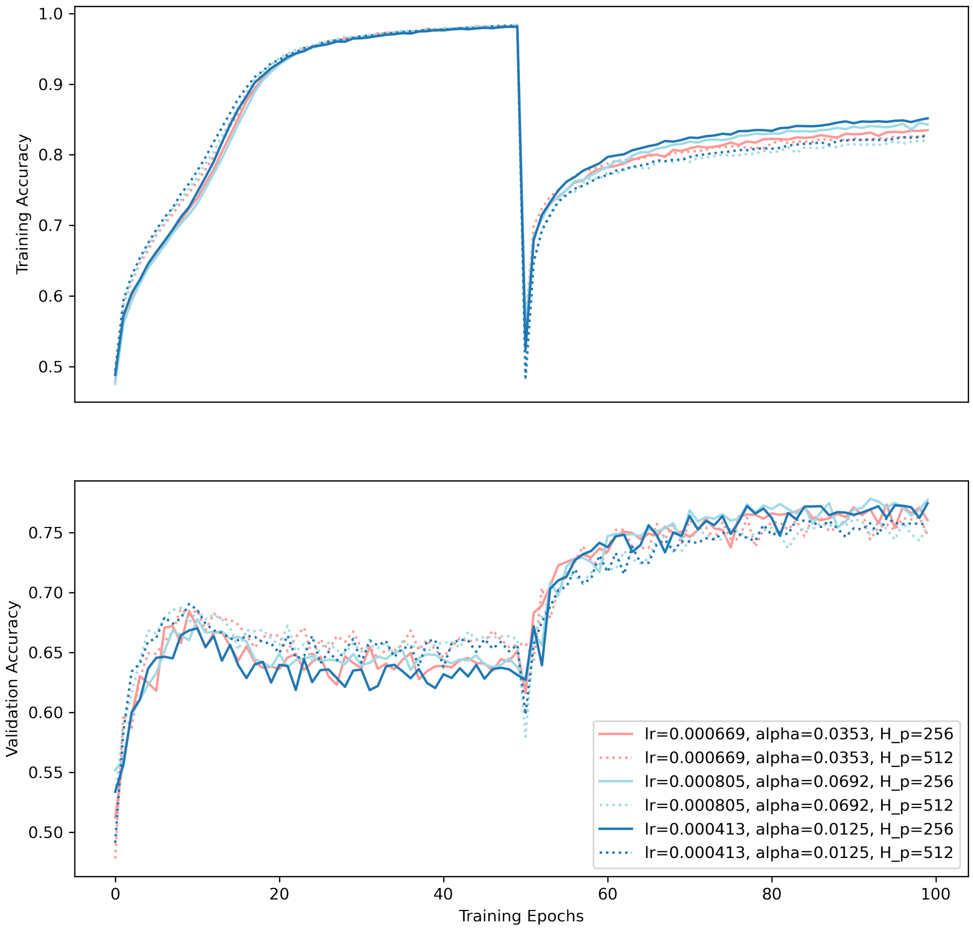

Figure 5: Effect of patch size on GridNet registration accuracy during two-stage training on the Maniatis dataset. Training (top) and validation (bottom) accuracy are plotted over time for GridNet models trained on patches of width $H_p = 256$ (solid lines) and $H_p = 512$ (dashed lines) under the three best-performing hyperparameter combinations found in Section 3.2 (Supplementary Table 1).

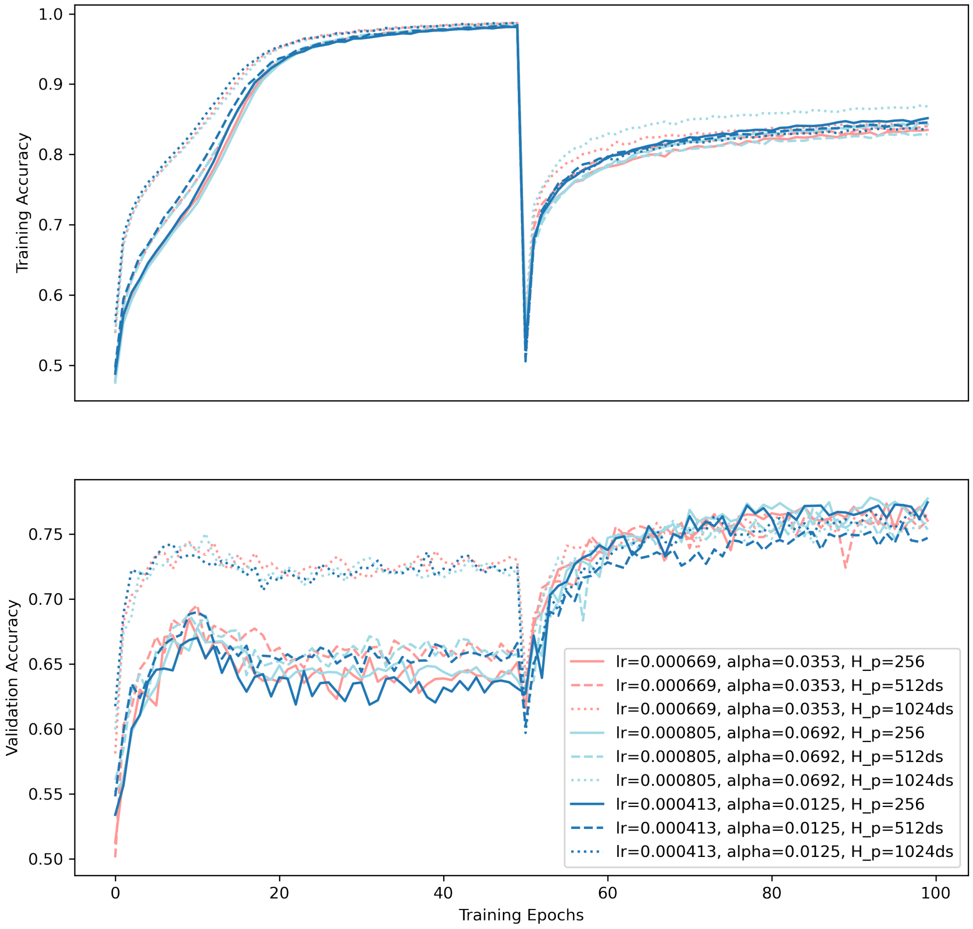

Figure 6: Effect of downsampled window side on GridNet registration accuracy during two-stage training on Maniatis dataset. Training (top) and validation (bottom) accuracy are plotted over time for GridNet models trained on patches sampled from windows of width 256 (solid), 512 (dashed), or 1024 pixels (dotted), then resized so that $H_p = 256$. Performance is shown under the three best-performing hyperparameter combinations found in Section 3.2 (Supplementary Table 1).

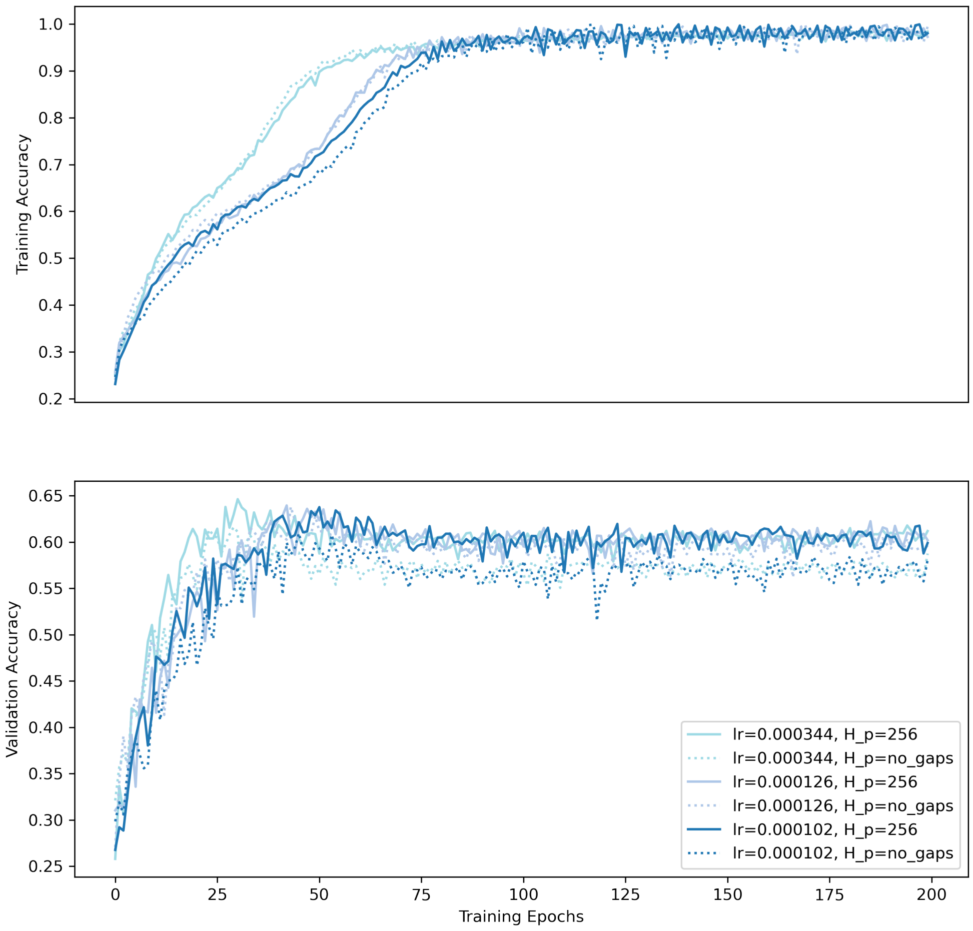

Figure 7: Effect of window size on Resnet-34-Seg registration accuracy during training on the Maniatis dataset. Training (top) and validation (bottom) accuracy are plotted over time for ResNet-34-Seg models trained on patches of either fixed width $H_p = 256$ (solid lines), or width chosen according to the center-center distance between regions of interest (dashed lines). This "no gap" strategy ensures that the full tissue area is covered by sampled patches without discontinuity or overlap. Image patches sampled in this manner are resized to 256 pixels by bicubic interpolation before input to the model. The comparison is carried out under the three best-performing hyperparameter combinations found in Section 3.2 (Supplementary Table 1).

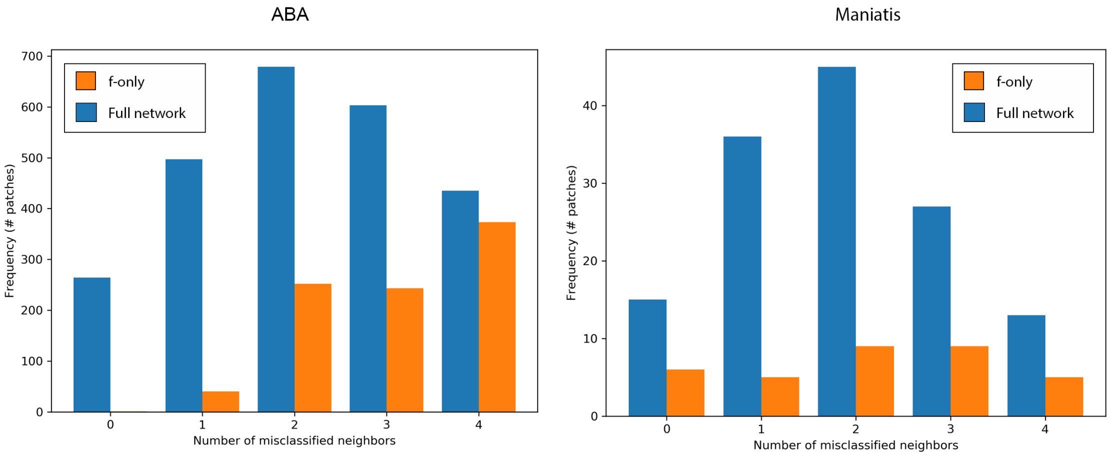

Figure 8: Frequency of misclassified patches in regions of mono-class tissue (all patches within unit distance are of same tissue type) conditional on the number of misclassified neighboring patches. Results are shown for both f-only (left) and full GridNet models (right) on both ABA (top) and Maniatis (bottom) datasets, using the best-performing models described in Section 3.2 and Supplementary Table 1 (ABA: lr=1.847e-4, $\alpha = 5.618e-2$; Maniatis: lr=6.685e-4, $\alpha = 3.527e-2$).
